# Efficacy and Safety of *Sophora flavescens* (Kushen) Based Traditional Chinese Medicine in the Treatment of Ulcerative Colitis: Clinical Evidence and Potential Mechanisms

**DOI:** 10.3389/fphar.2020.603476

**Published:** 2020-12-10

**Authors:** Mingjun Chen, Yuxuan Ding, Zhanqi Tong

**Affiliations:** ^1^ Department of Traditional Chinese Medicine, Second Medical Centre of Chinese PLA General Hospital, National Clinical Research Center for Geriatric Diseases, Beijing, China; ^2^ Chinese PLA Medical School, Beijing, China

**Keywords:** Sophora flavescens, ulcerative colitis, meta-analysis, herbal medicine, network pharmacology analysis, damp-heat accumulation syndrome

## Abstract

**Background:** Radix *Sophorae flavescentis* (Kushen), a Chinese herb, is widely used in the treatment of ulcerative colitis (UC) with damp-heat accumulation syndrome (DHAS) according to traditional Chinese medicine (TCM) theory.

**Objective:** The aim of this study was to illuminate the clinical efficacy and potential mechanisms of Kushen-based TCM formulations in the treatment of UC with DHAS.

**Materials and Methods:** A systematic literature search was performed in the PubMed, EMBASE, Chinese Biomedical Literature database, China National Knowledge Infrastructure database, Chongqing VIP Information database, and Wanfang database for articles published between January 2000 and July 2020 on randomized controlled trials (RCTs) that used Kushen-based TCM formulations in the treatment of UC with DHAS. A network pharmacology approach was conducted to detect the potential pathways of Kushen against UC with DHAS.

**Results:** Eight RCTs with a total of 983 subjects were included in the meta-analysis. Compared with the control subjects (5-aminosalicylic acid therapy), those who received Kushen-based TCM formulations for the treatment of UC showed a significantly higher clinical remission rate (RR = 1.20, 95% CI: [1.04, 1.38], *p* = 0.02) and lower incidence of adverse events (RR = 0.63, 95% CI [0.39, 1.01], *p* = 0.06). A component-target-pathway network was constructed, indicating five main components (*quercetin*, *luteolin*, *matrine*, *formononetin*, and *phaseolin*), three major targets (Interleukin-6, Myc proto-oncogene protein, and G1/S-specific cyclin-D1) and one key potential therapeutic pathway (PI3K-Akt signaling) of Kushen against UC with DHAS.

**Conclusion:** Kushen-based TCM formulations provide good efficacy and possess great potential in the treatment of UC. Large-scale and high-quality clinical trials and experimental verification should be considered for further confirmation of the efficacy of Kushen-based formulations.

## Introduction

Ulcerative colitis (UC) is a chronic inflammatory bowel disease that primarily occurs in the colon or rectum. The incidence of UC is generally higher in Caucasian populations, with the incidence rate among this group being as high as 70 per 100,000 individuals (Ordas et al., 2012; Sharma et al., 2018). However, it is important to note that the incidence of UC among East Asia populations has risen steadily in recent years given continuously improving living standards, with the incidence rate in Hong Kong and China reaching 24.5 per 100,000 individuals and 11.60 per 100,000 individuals, respectively (Ng et al., 2013; Ng et al., 2016). Although drugs such as 5-aminosalicylic acid, glucocorticoids, and tumor necrosis factor (TNF) blockers are commonly used in the treatment of UC, different problems exist, including recurrence, multiple adverse reactions, and poor effectiveness. At present, it is generally believed that the onset of UC is associated with factors such as gut dysbiosis, gut flora disorders, and immune dysfunction. Dysbiosis causes abnormal reactions between certain intestinal flora and the immune system, inducing or prolonging chronic bowel inflammation (Levy et al., 2015; Naganuma et al., 2016; Sandborn et al., 2017).

**FIGURE 1:**
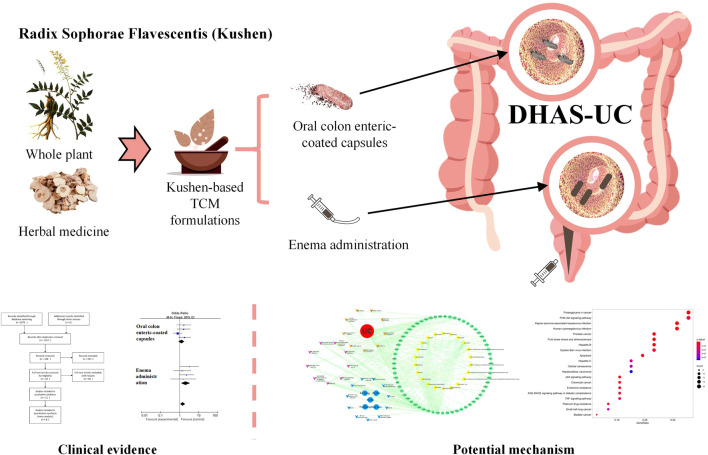
Clinical evidence and potential mechanism of Kushen for the treatment of (ulcerative colitis) UC with (damp-heat accumulation syndrome) DHAS.

Many herbal medicines have showed significant efficacy compared with 5-aminosalicylic acid therapy (5-ASA) (Langhorst et al., 2015; Naganuma et al., 2018; Naganuma, 2019). Based on the traditional Chinese medicine (TCM) theory, UC consists of several types of TCM syndromes, the most common of which (34.8%) being damp-heat accumulation syndrome (DHAS) (Ye et al., 2010). Radix *Sophorae flavescentis* (Kushen) is a monarch herb in TCM formulations (Kushen-based TCM formulations) used for the treatment of UC with DHAS which has achieved good effectiveness in clinical practice and effect studies (Tong et al., 2010; Tong et al., 2011; Gong et al., 2012; Fang et al., 2018; Chen et al., 2020). Kushen mainly contains the components *matrine* and *oxymatrine*, which are active ingredients against UC with significant inhibitory effects on invasive microbial populations such as *Escherichia coli* and *Proteus mirabilis,* as well as immunosuppressive effects against IL-1β and IL-17 in the intestinal epithelium (Lin et al., 2019; Xu et al., 2019; Ding et al., 2020). Based on these findings, meta-analysis and network pharmacology approaches were conducted to examine the published efficacies of Kushen-based TCM formulations compared to 5-ASA in the treatment of UC, detect and identify potential pathways of Kushen against UC with DHAS, and provide evidence-based scientific support for UC treatment in clinical practice (Figure 1).

## Data and Methods

### Inclusion and Exclusion Criteria for Studies

#### Selection of Studies

A systematic literature search was performed to identify randomized controlled trials (RCTs) that involved the use of Kushen-based TCM formulations alone or in combination with 5-ASA for the treatment of UC with DHAS. No exclusion criteria were defined for the publication language of the articles. Computer searches were performed in PubMed, EMBASE, Chinese Biomedical Literature (CBM) database, China National Knowledge Infrastructure (CNKI) database, Chongqing VIP Information (CQVIP) database, and Wanfang Database for the retrieval of relevant articles published from January 2000 up to July 2020. The search keywords we used included “UC” AND (“*Sophora flavescens*” OR “Radix *Sophorae flavescentis*” OR “kushen” OR “TCM”) AND (“randomized” OR “randomised” OR “RCT”), and the search strategy was confirmed after multiple preliminary searches.

### Inclusion and Exclusion Criteria

Studies were required to fulfill the following inclusion criteria to be part of the meta-analysis: 1) studies with a prospective randomized controlled trial design; 2) studies that included UC patients whose clinical presentations, colonoscopy results, and mucosal biopsy results follow the diagnostic criteria stated in the Third European Evidence-based Consensus on Diagnosis and Management of UC (Magro et al., 2017); and 3) studies that included patients who fulfilled the criteria for the diagnosis of UC with DHAS under the Consensus on the Diagnosis and Treatment of UC in TCM (2017) (Zhang et al., 2017). Furthermore, the following exclusion criteria were set: 1) non-RCTs; 2) studies that included subjects with a history of infectious colitis, Crohn’s disease or colon tumors, severe complications such as intestinal obstruction, intestinal perforation or toxic megacolon, or other severe systemic diseases; 3) studies in which the baseline data of the observational and control groups were different and non-comparable; 4) articles from which remission rates or adverse events could not be acquired; 5) studies with a sample size <50.

### Intervention

In the selected studies, the treatment regimen for the control group consisted of conventional 5-ASA for fever and pain relief. Interventions for the treatment group consisted of Kushen-based formulations in any form, including oral and rectal administration.

### Data Screening and Extraction

Two researchers (Dr. Ding and Dr. Chen) independently retrieved articles from the databases under the inclusion and exclusion criteria described above and extracted the following data from each article: authors’ names, date of publication, intervention method, the composition of TCM formulation, routes of administration, remission rate, and adverse drug reactions. Disagreements in the included studies were resolved by cross-checking and discussion between the two researchers, seeking the opinion of a third researcher (Prof. Tong), or contacting the authors as necessary.

### Assessment of the Risk of Bias of Included Studies

The risk of bias of the included RCTs in the aspects of randomization, allocation, and loss to follow-up were assessed following the Cochrane Handbook for Systematic Reviews of Interventions (Cumpston et al., 2019). Each risk of bias item was rated as “Yes” (low risk of bias), “No” (high risk of bias), or “Unclear” (lack of relevant information or unclear risk of bias). The two review authors (Dr. Ding and Dr. Chen) independently assessed the risk of bias in the included studies based on the following characteristics: randomized sequence generation, treatment allocation concealment, blinding, completeness of outcome data, selective outcome reporting, and other sources of bias. Disagreements between the review authors over the risk of bias in specific studies were resolved by discussion, with the involvement of a third review author (Prof. Tong) as needed.

### Types of Outcome Measures

The primary outcome indicator of the included studies was clinical remission after treatment, which was defined as a total Mayo score of ≤2, with no sub-score ≥1 and a rectal bleeding sub-score of 0. Alternatively, the reported clinical remission rates were assessed based on the diagnostic criteria. The secondary outcome was the clinical response, Chinese medical syndrome scores, Mayo (endoscopy) scores, and incidence of adverse events. Clinical response was evaluated in terms of basic or complete disappearance of clinical symptoms. Adverse events during the intervention period included infections that required treatment, hospitalization or surgery, or death.

### Statistical Analysis

The meta-analysis was performed with RevMan 5.3.5 (The Cochrane Collaboration) using the risk ratio (RR) and mean differences (MD). If heterogeneity (I^2^ > 50%) existed between different treatment groups in the included studies, the random-effects model was adopted for the calculation of overall mean differences and the corresponding 95% confidence intervals. Otherwise, calculations were performed using the fixed-effects model. Differences were considered statistically significant when *p* < 0.05.

### Mechanisms of Network Pharmacology of Kushen Against Ulcerative Colitis With Damp-Heat Accumulation Syndrome

All components in Kushen were retrieved from the TCM Systems Pharmacology (TCMSP) database (Ru et al., 2014). Candidate component properties with a drug-likeness (DL) index ≥0.18 and oral bioavailability (OB) ≥30%, were selected in the present study to explore the potential bioactive components of Kushen (Wang P. et al., 2019; Guo et al., 2019). The corresponding targets of the bioactive components in Kushen were retrieved with further importation into the DrugBank database, and the data for UC-related target genes were identified from the GeneCards database (Gan et al., 2019). Target genes with a relevance score ≥1 were screened to be notable UC-associated expression targets (Wang et al., 2020). Then, the TCM syndrome of DHAS, including main TCM symptoms such as, “Fa Re” (Fever), “Fu Tong” (Abdominal Pain), “Fu Xie” (Diarrhea), “Fu Zhang” (Dilation), and “Kou Gan” (Xerostomia), were submitted to the SymMap database for transcription of their corresponding target genes (Wang T. et al., 2019).

The Component-Target (C-T) network of Kushen against UC/DHAS was established by using the Cytoscape v.3.7.0 software (Reimand et al., 2019). The features of the degrees were recognized as a critical parameter of the “Network Analyzer” tool for screening core nodes in the C-T network. Additionally, the common set of targets between Kushen and UC-related/DHAS-related targets derived from the previous identification were submitted to the STRING database for protein-interaction network mapping, and predicted direct and functional target genes in medium confidence (PPI score ≥0.4) were collected to establish PPI networks of UC-related and DHAS-related targets (Martin et al., 2010; Wang P. et al., 2019).

All the candidate components and genes were further assessed via pathway enrichment analysis (Kyoto Encyclopedia of Genes and Genomes, KEGG). The system R3.5.0 was utilized to detect the potential pathways of candidate genes of UC-related and DHAS-related targets. The top 20 terms of both highly enriched pathways were imported into Cytoscape for visualization of the Component-Target-Pathway (C-T-P) network of Kushen against UC/UC with DHAS. The “Network Analyzer” function of Cytoscape was also employed for exploration of the key targets, components, and pathways with the greatest degrees.

## Results

### Results of Literature Search

**FIGURE 2:**
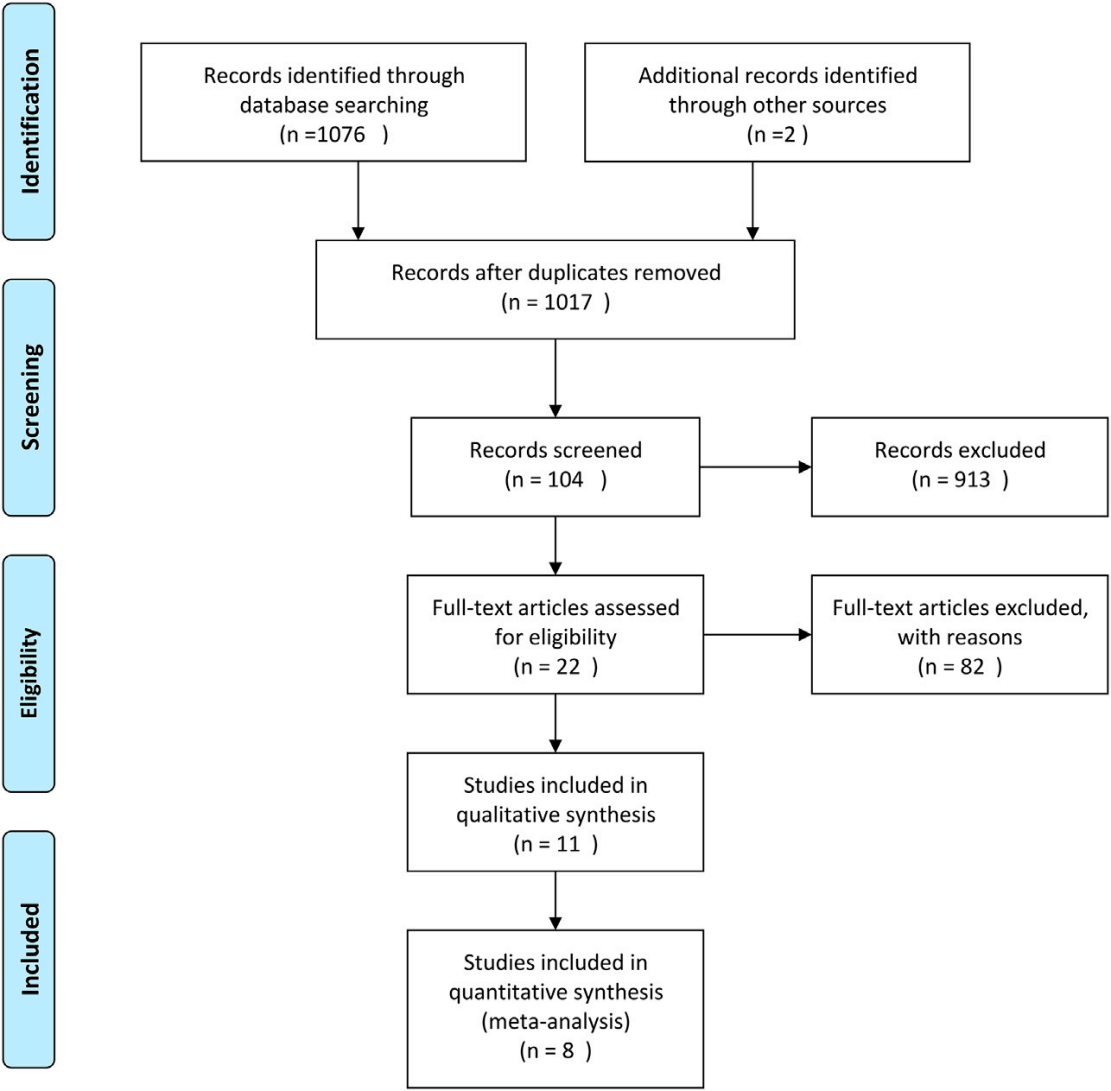
Flow diagram of the literature screening and study selection.

A total of 1,108 articles were returned by the preliminary search. The title, abstract, and introduction of each article were read to exclude reviews, case reports, animal experiments, duplicate articles, and other articles that did not fulfill the inclusion and exclusion criteria. After the full text of the 22 articles that were preliminarily included had been read, eight published RCTs were ultimately included in the meta-analysis (Tong et al., 2010; Qu, 2011; Tong et al., 2011; Gong et al., 2012; He et al., 2012; Chen and He, 2014; Zhang et al., 2015; Zhao, 2017). The flow diagram and results of the literature screening and study selection process are shown in Figure 2 and Table 1.

**TABLE 1:** Characteristics of included studies.

Study	Sample size		Intervention measures		Administration	Duration (Western medicine/TCM)	No. of adverse events (treatment group/control group)	No. of withdrawals (treatment group/control group)
	Treatment group	Control group	Treatment group	Control group				
Zhao (2017)	60	60	Compound Kushen Enteric Capsule	5-ASA	Oral	Mild, moderate, severe UC/Damp-heat accumulation syndrome	0/6	—
Zhang et al. (2015)	45	40	Kushen*-*based Enema Solution+5-ASA	5-ASA	Enema	Mild, moderate UC/Damp-heat accumulation syndrome	3/10	—
Chen and He (2014)	48	48	Retention Enema with TongGuan Solution	5-ASA	Enema	Chronic UC/ Damp-heat accumulation syndrome	0	—
He et al. (2012)	30	30	Kushen*-*based Enema Solution	5-ASA	Enema	Mild, moderate, recurrent UC/ Damp-heat accumulation syndrome	0/1	—
Gong et al. (2012)	240	80	Fufangkushen Colon-coated Capsule	5-ASA	Oral	Mild, moderate, severe UC/Damp-heat accumulation syndrome	38/10	35/18
Tong et al. (2011)	120	40	Composite Sophora Colon-Soluble Capsules	5-ASA	Oral	Mild, moderate, severe UC/Damp-heat accumulation syndrome	2/2	20/4
Qu (2011)	30	28	Baiji Kusen Decoction+5-ASA	5-ASA	Enema	Mild, moderate, severe UC/Damp-heat accumulation syndrome	0	—
Tong et al. (2010)	42	42	Composite Sophora Colon-Soluble Capsules	5-ASA	Oral	Mild, moderate, severe UC/Damp-heat accumulation syndrome	2/2	1/1

5-ASA, 5-aminosalicylic acid therapy.

### General Data of Included Studies

**FIGURE 3:**
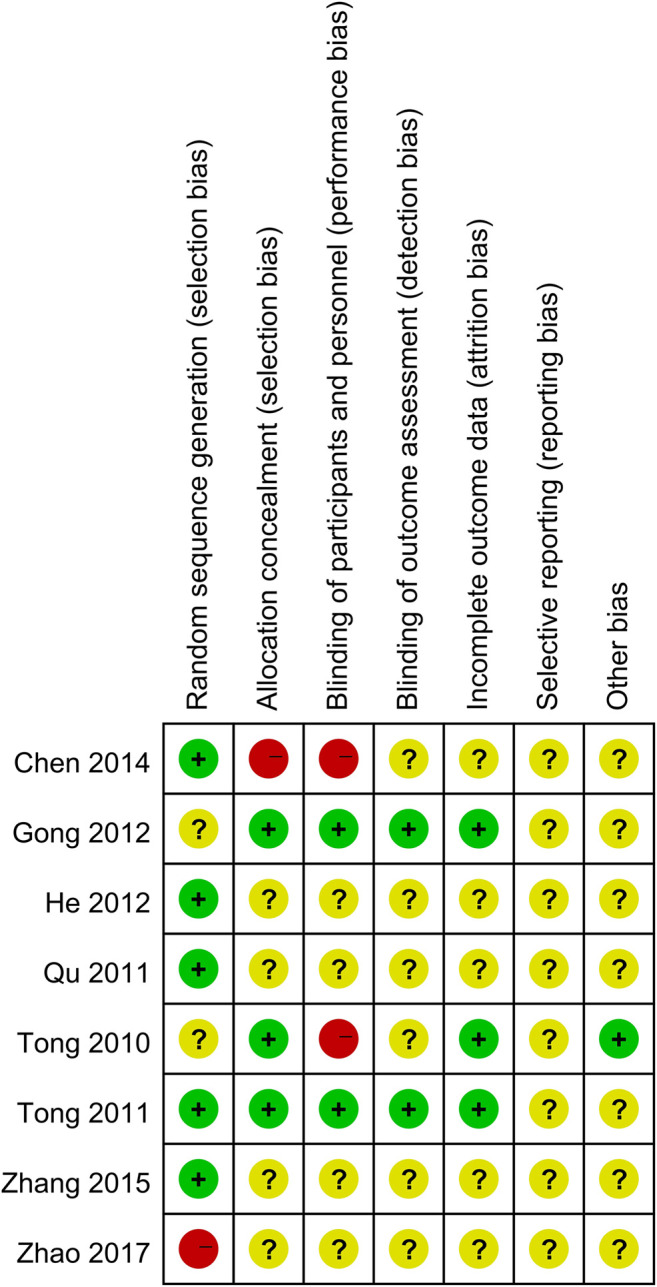
Risk of bias summary.

A total of 983 subjects were included in the selected RCTs. There were no differences in the baseline data of the treatment and control groups. The treatment regimens for the control and treatment groups were conventional Western drugs (5-ASA) and Kushen-based TCM formulations, respectively. Among all included trials, Kushen-based TCM formulations were prescribed with direct action on the local target tissues of the colon by oral administration of colon enteric-coated capsules (4 trials) or enema administration (4 trials). The general data of the included studies and the components and types of the TCM formulations used are detailed in Tables 1, 2. Subjects were randomized in all eight studies, and the strict enforcement of allocation concealment to ensure proper randomization was mentioned in three reports (Tong et al., 2010; Tong et al., 2011; Gong et al., 2012). Figure 3 shows the results of the risk of bias assessment.

**TABLE 2:** Data regarding Kushen-containing TCM formulations.

Study	TCM formulations	Components
Zhao (2017)	Compound Kushen Enteric capsule	Kushen (radix sophorae flavescentis), Diyu (the root of *Sanguisorba officinalis*), Qingdai (extract of leaves of indigo-bearing plants), Baiji (tuber of *Bletilla striata*), raw gancao (the root of *Glycyrrhiza uralensis*)
Zhang et al. (2015)	Kushen*-*based enema Solution + M	Kushen, raw Diyu, Xianhecao (aboveground parts of *Agrimonia pilosa*), Baimaogen (Rhizoma imperatae), Baiji, Huangqin (the root of *Scutellaria baicalensis*), dried alum, borneol
Chen and He (2014)	Retention enema with TongGuan solution	Kushen, Huangbai (Phellodendron bark), Baiji, alum, Xueyutan (carbonized hair), Chuanxinlian (aerial parts of *Andrographis paniculate*), Qingdai, Sanqi (the root of *Panax notoginseng*), Ercha (heartwood extract of catechu), Muxiang (the root of *Saussurea costus*)
He et al. (2012)	Kushen*-*based enema solution	Kushen, Huangbai, Diyu, Baiji, powdered Sanqi, Xileisan (a compound TCM herbal preparation)
Gong et al. (2012)	Fufangkushen Colon-coated capsule	Kushen, diyu, qingdai, baiji, raw gancao
Tong et al. (2011)	Composite Sophora Colon-soluble capsules	Kushen, Diyu, Qingdai, Baiji, raw Gancao
Qu (2011)	Baiji Kusen decoction + S	Kushen, baiji, baizhu (rhizome of *Atractylodes macrocephala*), baishao (the root of *Peonia lactiflora*)
		Zhike (immature bitter orange peel), Yingsuke (opium poppy husk), Paojiang (roasted ginger)
Tong et al. (2010)	Composite Sophora Colon-soluble capsules	Kushen, Diyu, Qingdai, Baiji, raw Gancao

### Clinical Remission Rates of the Treatment and Control Groups

**FIGURE 4:**
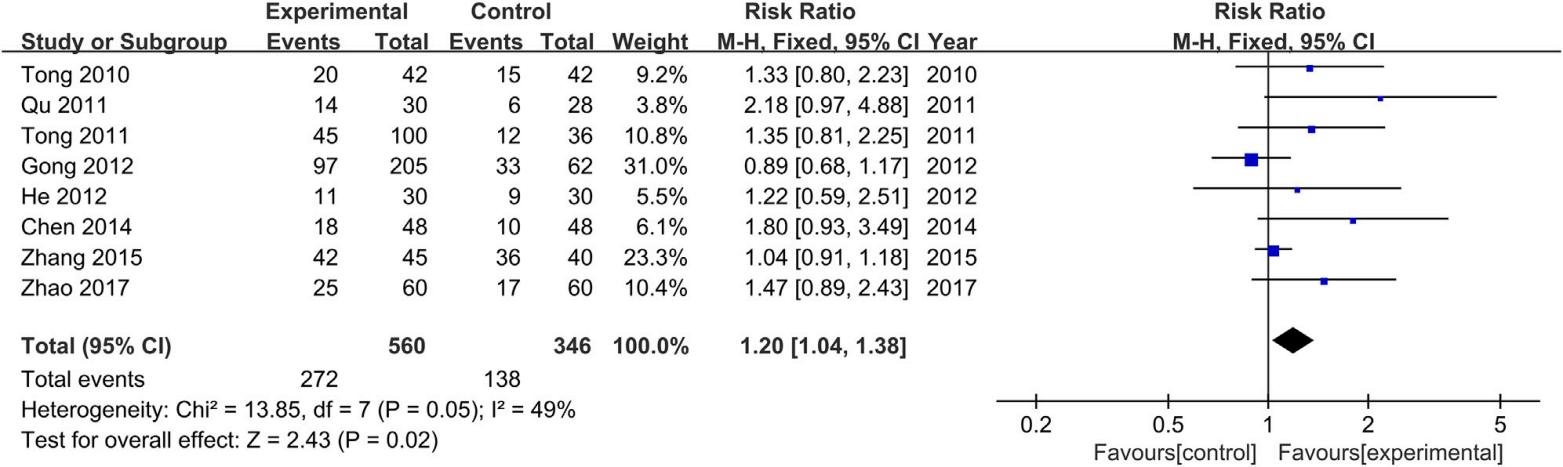
Forest plot for the meta-analysis of clinical remission rates.

The comparisons of clinical remission rates between the treatment and control groups were reported in all the included studies (Tong et al., 2010; Qu, 2011; Tong et al., 2011; Gong et al., 2012; He et al., 2012; Chen and He, 2014; Zhang et al., 2015; Zhao, 2017). There was no significant heterogeneity among the various studies (Chi^2^ = 13.86, *p* = 0.05, I^2^ = 49%). Results obtained with the fixed effects model indicate that the efficacy achieved in the treatment groups was significantly higher than those of the control groups (RR = 1.20, 95% CI [1.04, 1.38], *p* = 0.02, Figure 4).

### Clinical Response Rates of the Treatment and Control Groups

**FIGURE 5:**
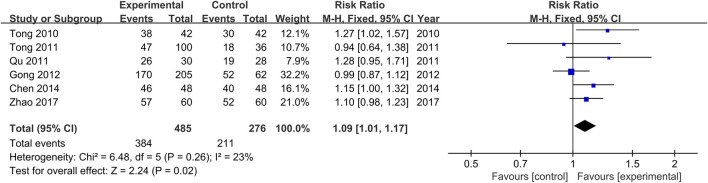
Forest plot for the meta-analysis of Clinical response rates.

The comparisons of clinical response rates between the treatment and control groups were reported in all the included studies (Tong et al., 2010; Qu, 2011; Tong et al., 2011; Gong et al., 2012; Chen and He, 2014; Zhao, 2017). There was no significant heterogeneity among the various studies (Chi^2^ = 6.48, *p* = 0.26, I^2^ = 23%). Results obtained with the fixed effects model indicate that clinical response rates of the treatment groups were significantly higher than those of the control groups (RR = 1.09, 95% CI [1.01, 1.17, *p* = 0.02, Figure 5).

### Chinese Medical Syndrome Scores of the Treatment and Control Groups

**FIGURE 6:**
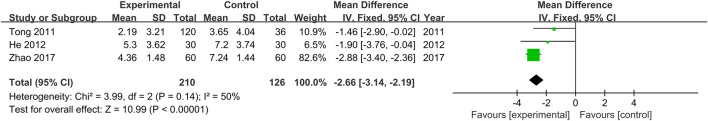
Forest plot for the meta-analysis of Chinese medical syndrome scores.

Chinese medical syndrome scores were provided in three studies (Tong et al., 2011; He et al., 2012; Zhao, 2017). As no heterogeneity was found (Chi^2^ = 3.99, *p* = 0.14), the fixed effects model was employed. Results indicate that Chinese medical syndrome scores of the treatment groups were lower than those of the control groups (MD = −2.66, 95% CI [−3.14, −2.19, *p* < 0.00001, Figure 6).

### Mayo (Endoscopy) Scores of the Treatment and Control Groups

**FIGURE 7:**
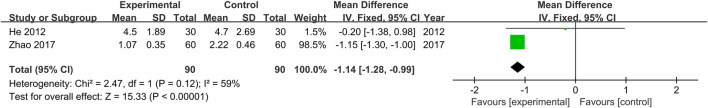
Forest plot for the meta-analysis of Mayo (endoscopy) scores.

Mayo (endoscopy) scores were provided in two studies (He et al., 2012; Zhao, 2017). No heterogeneity was found (Chi^2^ = 2.47, *p* = 0.12) across the studies, and results obtained with the fixed effects model indicate that Mayo (endoscopy) scores of the treatment groups were lower than those of the control groups (MD = −1.14, 95% CI [−1.28, −0.99, *p* < 0.00001, Figure 7).

### Adverse Events of the Treatment and Control Groups

**FIGURE 8:**
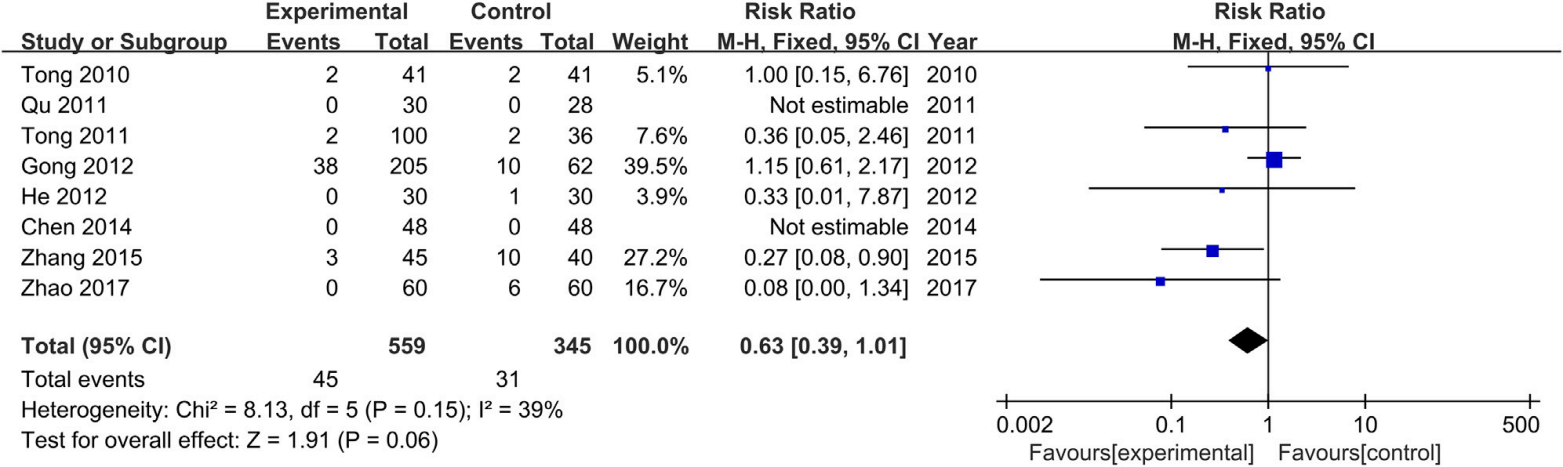
Forest plot for the meta-analysis of adverse drug reaction rates.

The occurrence or non-occurrence of adverse events after treatment was reported in all studies. Non-occurrence of adverse drug reactions was reported in two studies (Qu, 2011; Chen and He, 2014), and a total of 72 adverse events were reported in the other six studies (Tong et al., 2010; Tong et al., 2011; Gong et al., 2012; He et al., 2012; Zhang et al., 2015; Zhao, 2017), with 44 occurring in the treatment groups and 28 in the control groups. Adverse events of the treatment groups included nausea, fatigue, abdominal pain, pain around the anus, indigestion, drug prototype discharge, pyrexia, mild indigestion, and menstrual disorders. Adverse events of the control groups included fatigue, insomnia, nausea and vomiting, pruritus, and a mild elevation of ALT. Severe adverse events were not reported in all studies. Furthermore, there was no significant heterogeneity among the various studies (Chi^2^ = 8.13, *p* = 0.15, I^2^ = 39%). Results obtained with the fixed-effects model indicate that the adverse drug reaction rate of the treatment groups was lower than that of the control groups (RR = 0.63, 95% CI [0.39, 1.01], *p* = 0.06, Figure 8).

### Analysis of the Mechanisms of Network Pharmacology of Kushen Against Ulcerative Colitis With Damp-Heat Accumulation Syndrome

**FIGURE 9:**
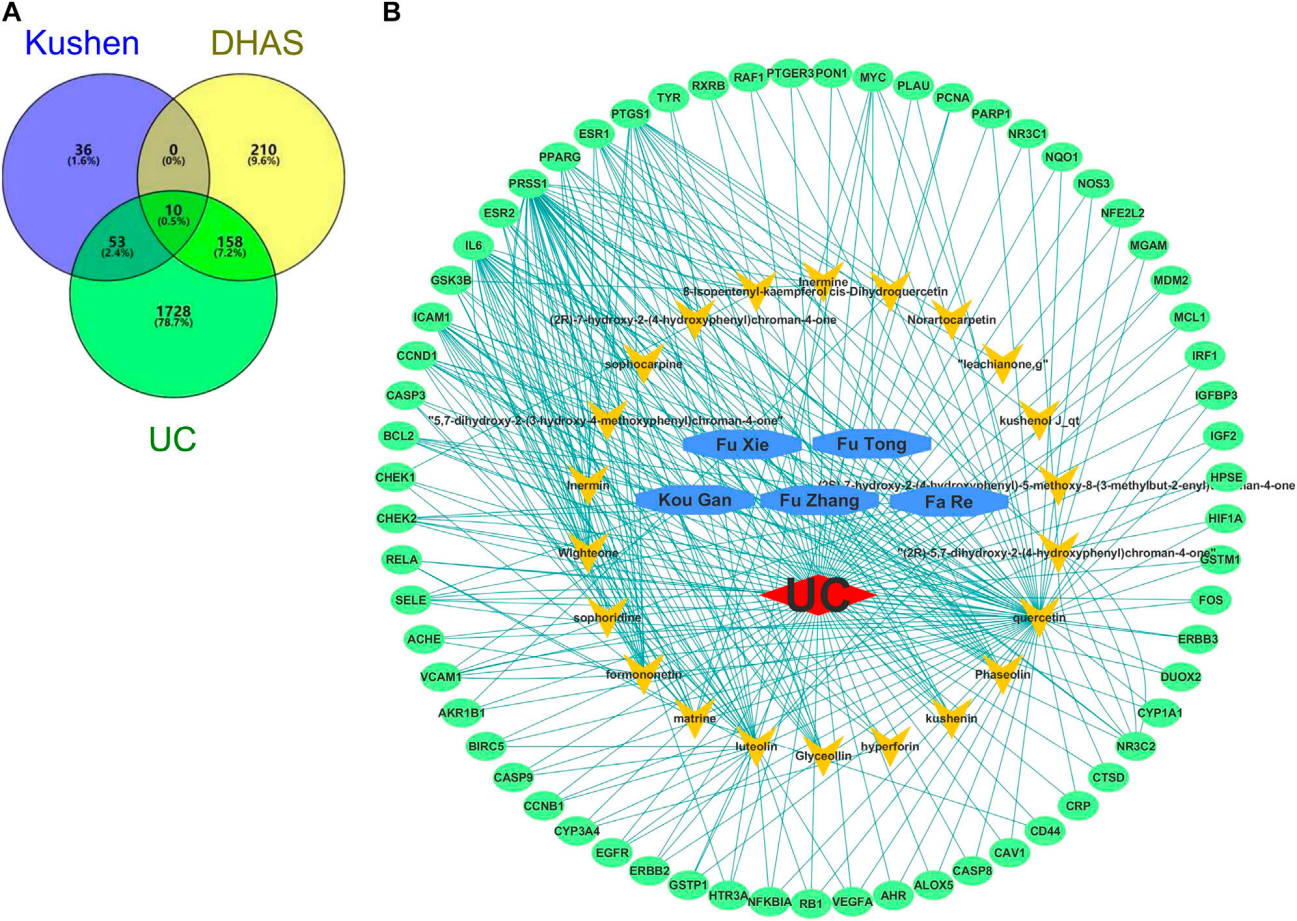
Overlapping targets of Kushen against (ulcerative colitis) UC and (damp-heat accumulation syndrome) DHAS related genes. **(A)** The number of common sets of targets between Kushen with UC and DHAS related targets were shown in the Venn diagram. There were 99 targets related to Kushen, 378 targets related DHAS, and 1949 targets related to UC. UC-related targets of Kushen shared 63 common targets, and DHAS shared 10 targets simultaneously. **(B)** The C-T network of Kushen against UC and DHAS-related targets containing 91 nodes and 292 edges. The yellow vees represent components of Kushen, the blue octagons represent the five TCM symptoms of UC with DHAS, the red diamond represents the disease UC, and the green ellipses represent the targets.

A total of 22 candidate bioactive components from Kushen were obtained from the TCMSP database (Table 3). About 201 component-target relationship data were detected from the Drugbank database (Supplementary Table S1), and 1,949 human target genes associated with UC were collected from the GeneCards database (relevance score ≥1, Supplementary Table S2). After the intersection, 63 consensus genes were found as potential therapeutic targets of Kushen against UC. Simultaneously, 463 human genes related to UC with DHAS TCM symptoms were retrieved from the SymMap database (Supplementary Table S3). Finally, 10 potential therapeutic targets of the common set of Kushen targets and UC with DHAS targets, together with the previous 63 consensus targets of Kushen against UC, were submitted to Cytoscape for the C-T network establishment (Figures 9A).

**TABLE 3:** Chemical properties of the components of Kushen with OB ≥ 30% and a DL index ≥0.18.

Parameter	Min	Max	Mean	Std. Deviation
OB	32.04	97.27	53.8624	13.66713
DL	0.18	0.76	0.3582	0.17403
MW	244.37	580.59	311.2391	91.10597
AlogP	−0.87	8.62	1.9693	1.65912
Hdon	0	7	1.89	1.910
Hacc	3	14	5.07	2.742

OB, Oral bioavailability; DL, Drug-likeness; MW, Molecular weight; AlogP, Partition coefficient between octanol and water; Hdon, hydrogen-bond donors; Hacc, hydrogen-bond acceptors.

**FIGURE 10:**
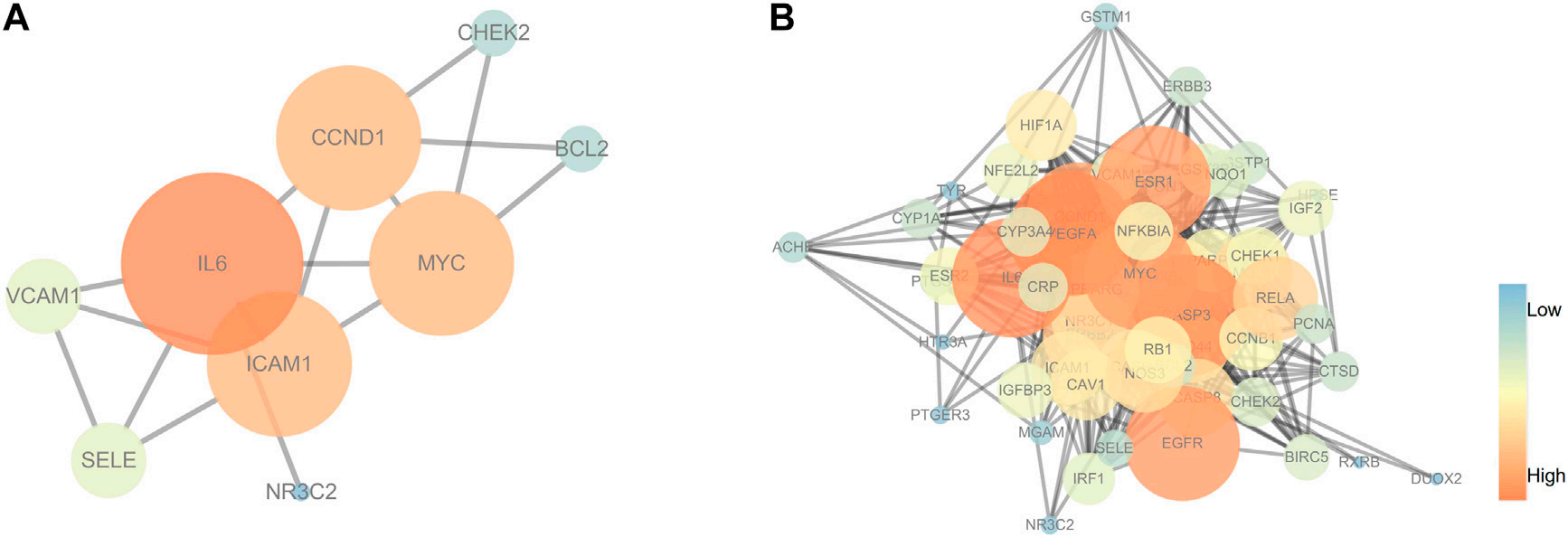
PPI networks of (ulcerative colitis) UC and UC with (damp-heat accumulation syndrome) DHAS related targets. **(A)** The PPI network of target genes from Kushen against UC with DHAS related targets. **(B)** The PPI network of target genes from Kushen against UC-related targets. The ellipse nodes represented targets, and the colors of nodes were illustrated from blue to orange in descending order of degree values.

The C-T network contained 91 nodes (22 components, 63 gene targets, and six disease labels) and 292 edges, indicating the component-target interactions of Kushen against UC/UC with DHAS TCM symptoms (Figures 9B). Five main bioactive components were identified to occupy a crucial position in this network, with node degrees higher than 10, including *quercetin*, *luteolin*, *matrine*, *formononetin*, and *phaseolin* (Table 4). Meanwhile, the 63 UC-related targets and 10 UC with DHAS TCM symptoms targets regulated by Kushen were submitted to the STRING database for generation of the protein interactions and construction of PPI network, where 61 UC-related targets and nine DHAS-related targets were predicted with medium confidence with a calculated interaction confidence score ≥0.4. New PPI networks were constructed after the DHAS and UC targets were separately submitted into Cytoscape (Figures 10A,B). Among the DHAS-related targets of Kushen, interleukin-6 (IL-6) was identified as the major target with top node degrees via Network Analyzer. Meanwhile, the top three target genes of Kushen against UC, detected with the greatest degrees, were vascular endothelial growth factor A (VEGFA), Caspase-3 (CASP3), and IL-6. The detailed results are shown in Tables 5, 6.

**TABLE 4:** Top five components of Kushen in degrees from Network Analyzer.

Molecular ID	Molecule name	OB (%)	DL	Degree
MOL000098	*Quercetin*	46.43	0.28	69
MOL000006	*Luteolin*	36.16	0.25	31
MOL005944	*Matrine*	63.77	0.25	13
MOL000392	*Formononetin*	69.67	0.21	12
MOL000456	*Phaseolin*	78.2	0.73	10

**TABLE 5:** The topological parameters of UC with DHAS targets of Kushen from the String database (Top 10 in degrees).

Name	Description	Degree	Average shortest path length	Betweenness centrality	Closeness centrality
IL6	Interleukin-6	6	1.25	0.39285714	0.8
MYC	Myc proto-oncogene protein	5	1.375	0.19642857	0.72727273
CCND1	G1/S-specific cyclin-D1	5	1.375	0.19642857	0.72727273
ICAM1	Intercellular adhesion molecule 1	5	1.375	0.14285714	0.72727273
SELE	E-selectin	3	1.875	0	0.53333333
VCAM1	Vascular cell adhesion protein 1	3	1.875	0	0.53333333
BCL2	Apoptosis regulator Bcl-2	2	2.125	0	0.47058824
CHEK2	Serine/threonine-protein kinase Chk2	2	2.125	0	0.47058824
NR3C2	Mineralocorticoid receptor	1	2.125	0	0.47058824

**TABLE 6:** The topological parameters of UC targets of Kushen from the String database (Top 10 in degrees).

Name	Description	Degree	Average shortest path length	Betweenness centrality	Closeness centrality
VEGFA	Vascular endothelial growth factor A	45	1.26229508	0.10527857	0.79220779
CASP3	Caspase-3	44	1.27868852	0.07339591	0.78205128
IL6	Interleukin-6	43	1.29508197	0.10311185	0.7721519
EGFR	Epidermal growth factor receptor	41	1.32786885	0.04501933	0.75308642
ESR1	Estrogen receptor	40	1.3442623	0.05122988	0.74390244
MYC	Myc proto-oncogene protein	40	1.3442623	0.04340003	0.74390244
CCND1	G1/S-specific cyclin-D1	38	1.37704918	0.02824009	0.72619048
ERBB2	Receptor tyrosine-protein kinase erbB-2	32	1.47540984	0.02728821	0.67777778
FOS	Proto-oncogene c-Fos	31	1.49180328	0.03609432	0.67032967
MDM2	E3 ubiquitin-protein ligase Mdm2	29	1.54098361	0.01135205	0.64893617

**FIGURE 11:**
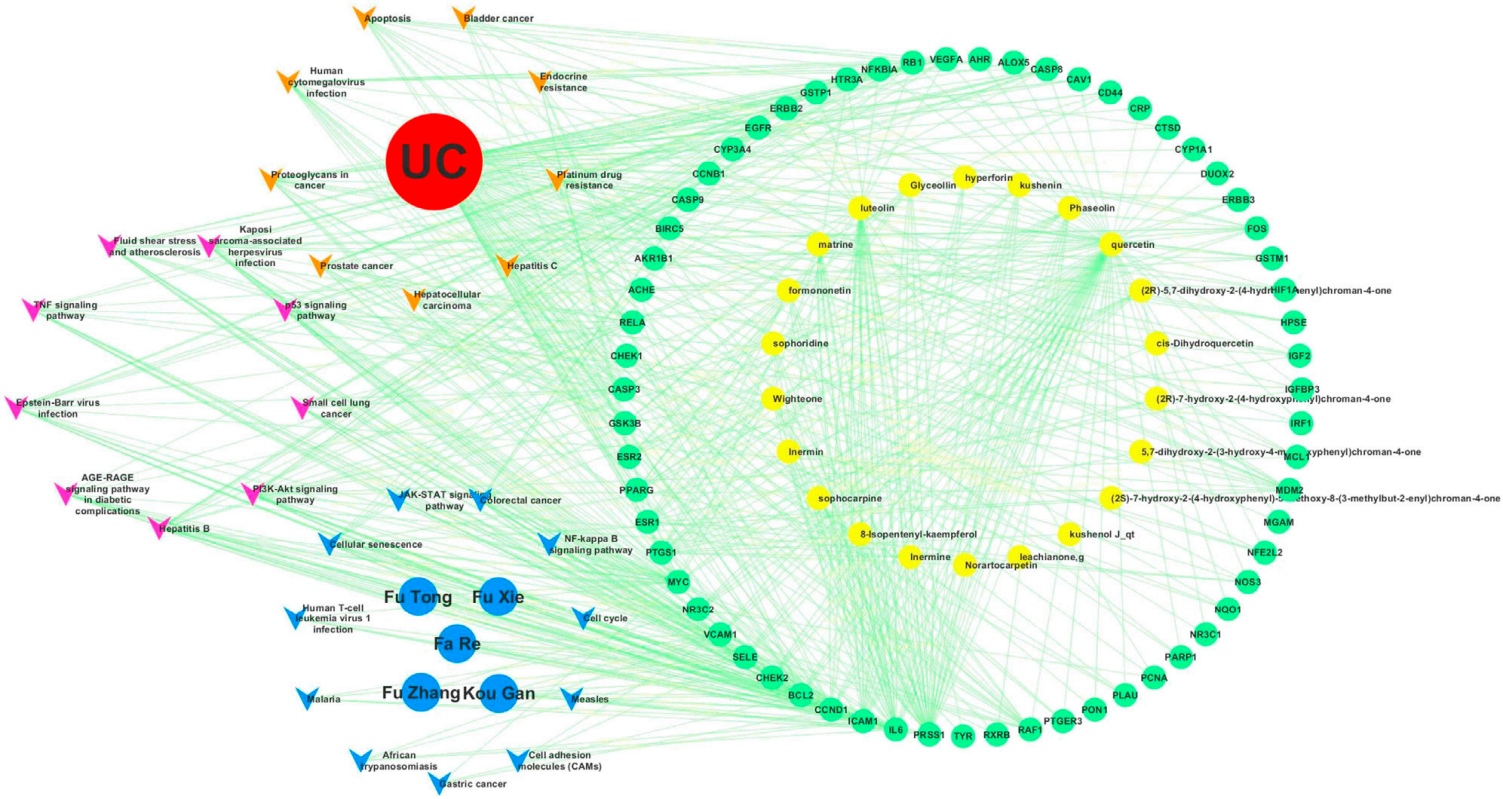
Components-targets-pathways network of Kushen. The top 20 enriched KEGG pathways of (ulcerative colitis) UC and UC with (damp-heat accumulation syndrome) DHAS were integrally submitted to Cytoscape for Kushen C-T-P network constructions with nine common pathways of UC and DHAS, 11 unique UC-related pathways, and 11 unique DHAS-related pathways. The yellow, pink, and blue vees represent pathways of unique UC-related pathways, common pathways, and unique DHAS-related pathways, respectively. The red, blue, yellow, and green ellipses represent the disease UC, the TCM symptoms of DHAS, components of Kushen, and targets of Kushen, respectively.

KEGG pathway enrichment analyses were performed using R3.5.0. Among the 103 enriched KEGG pathways of UC-related targets that met the requirements of Adjust *p*-Value < 0.05, the top 10 enriched pathways with higher Gene Ratio values were shown in Table 7. The top enriched pathways were further integrated into a Components-Targets-Pathways network analysis (Figure 11), and *quercetin* (degree = 69, component), IL6 (degree = 44, gene), PRSS1 (degree = 44, gene), CCND1 (degree = 37, gene), *luteolin* (degree = 31, component), BCL2 (degree = 30, gene), ICAM1 (degree = 30, gene), MYC (degree = 29, gene), PI3K-Akt signaling pathway (degree = 20, pathway), and Kaposi sarcoma-associated herpes virus infection (degree = 19, pathway) were indicated to be the top 10 core nodes of components, target genes, and pathways (Supplementary Table S4). The PI3K-Akt signaling pathway has appeared in the common set of UC/UC with DHAS with greatest degrees in the pathway and was inferred as the key potential therapeutic pathway of Kushen against UC with DHAS.

**TABLE 7:** Detailed information of Kushen-related KEGG pathways against UC targets (Top 10 in Gene Ratio).

ID	Term	Gene Ratio	Count	Related targets	Adjust *p*-value
hsa05205	Proteoglycans in cancer	16/63	16	CASP3, CAV1, CCND1, CD44, EGFR, ERBB2, ERBB3, ESR1, HIF1A, HPSE, IGF2, MDM2, MYC, PLAU, RAF1, VEGFA	1.47E−10
hsa04151	PI3K-Akt signaling pathway	16/63	16	BCL2, CASP9, CCND1, EGFR, ERBB2, ERBB3, GSK3B, IGF2, IL6, MCL1, MDM2, MYC, NOS3, RAF1, RELA, VEGFA	7.51E−08
hsa05167	Kaposi sarcoma-associated herpesvirus infection	15/63	15	CASP3, CASP8, CASP9, CCND1, FOS, GSK3B, HIF1A, ICAM1, IL6, MYC, NFKBIA, RAF1, RB1, RELA, VEGFA	3.65E−10
hsa05163	Human cytomegalovirus infection	15/63	15	CASP3, CASP8, CASP9, CCND1, EGFR, GSK3B, IL6, MDM2, MYC, NFKBIA, PTGER3, RAF1, RB1, RELA, VEGFA	3.40E−09
hsa05215	Prostate cancer	13/63	13	BCL2, CASP9, CCND1, EGFR, ERBB2, GSK3B, GSTP1, MDM2, NFKBIA, PLAU, RAF1, RB1, RELA	4.80E−11
hsa05418	Fluid shear stress and atherosclerosis	13/63	13	BCL2, CAV1, FOS, GSTM1, GSTP1, ICAM1, NFE2L2, NOS3, NQO1, RELA, SELE, VCAM1, VEGFA	1.32E−09
hsa05161	Hepatitis B	13/63	13	BCL2, BIRC5, CASP3, CASP8, CASP9, FOS, IL6, MYC, NFKBIA, PCNA, RAF1, RB1, RELA	5.27E−09
hsa05169	Epstein-Barr virus infection	13/63	13	BCL2, CASP3, CASP8, CASP9, CCND1, CD44, ICAM1, IL6, MDM2, MYC, NFKBIA, RB1, RELA	4.61E−08
hsa05206	MicroRNAs in cancer	13/63	13	BCL2, CASP3, CCND1, CD44, EGFR, ERBB2, ERBB3, MCL1, MDM2, MYC, PLAU, RAF1, VEGFA	3.19E−06
hsa04210	Apoptosis	12/63	12	BCL2, BIRC5, CASP3, CASP8, CASP9, CTSD, FOS, MCL1, NFKBIA, PARP1, RAF1, RELA	8.34E−09

## Discussion

UC is a disease featuring immunological disorders in the intestinal tract caused by chronic intestinal inflammation, characterized by recurring episodes of inflammation and a relatively long disease course. It is currently believed that a key factor in the onset and development of UC is immune dysfunction in the intestinal mucosa, which was widely recognized as being associated with multiple cellular immune responses and dysbiosis (Eisenstein, 2018). However, conventional therapies might lead to poor effects on multiple pro-inflammatory targets, pathways, and the safety of UC patients; thus, in recent decades, plenty of Chinese herbs were employed for the treatment of intractable UC (Chudnovskiy et al., 2016; Cao et al., 2019).

Based on the TCM theory, UC consists of several types of TCM syndromes, most of which (34.8%) are for the DHAS, and Kushen (Radix *Sophorae flavescentis*) is a standard herb for the treatment of UC with DHAS (Ye et al., 2010). DHAS, a standard TCM syndrome (“Zheng” in Chinese), is defined as the accumulation of damp-heat TCM pathogen in the large intestine, ultimately leading to the obstruction of internal transportation (Lu and Cong, 2012; Chen et al., 2018). DHAS is derived from the conversion of external pathological substances into moisture and heat, resulting in colitis and mucosal injury when accumulated in the gastrointestinal tract (Ye et al., 2010). DHAS is therefore determined as a biased constitution that needs to be eliminated and regulated for the successful treatment of UC (Wang et al., 2019a).

Kushen is a kind of Chinese herbal medicine commonly used to treat UC with DHAS that exerts extensive influence in anti-inflammatory, immunosuppression, and antibacterial functions (Holleran et al., 2020). Studies have shown that *matrine*, a major component of Kushen, possesses broad-spectrum antibacterial effects. When used *in vitro*, Kushen can inhibit *E. coli*, *P. mirabilis*, and *Aerobacter* species, with its inhibitory effects being superior compared to the effects of chloramphenicol. Other experiments have shown that Kushen used *in vivo* can significantly reduce the number of invasive microbial populations in the intestines of mice, thereby lowering animal mortality caused by *E. coli* and *Clostridium perfringens* type B. Besides, Kushen was validated to inhibit NK-1R expression, leading to a reduced secretion of inflammatory cytokines such as IL-1β and IL-8 in cells and the promotion of IFN-γ synthesis (Holleran et al., 2020; Ding et al., 2020). Consequently, this produces certain regulatory effects on abnormal immune responses in the intestinal mucosa. These findings suggest that Kushen-based TCM formulations provide an effective treatment by improving immune dysfunction in the intestinal mucosa, cell immunity, and intestinal flora, which are key links in the pathogenesis of UC with DHAS.

In the present meta-analysis, a total of eight studies were included, among which three were multi-center clinical studies. Kushen-based TCM formulations were delivered site-specifically to the disease focus of UC directly in all included trials (four studies utilized oral colon enteric-coated capsules, four studies utilized enema administration). Results of the analysis showed that Kushen-based TCM formulations used alone or in combination with 5-ASA for the treatment of UC with DHAS led to a significantly higher clinical remission rate and lower incidence of adverse events in the treatment groups than in the control groups.

However, among the eight studies analyzed in this work, it should be noted that only three had clearly stated the enforcement of blinding, allocation concealment, and the reasons for subject withdrawal. Other articles were of lower quality as blinding and subject withdrawal had not been reported. Moreover, the result of clinical remission rates showed relatively high heterogeneity (*p* = 0.05, I^2^ = 49%), which may be attributed to several reasons. First, clinical diagnosis and treatment by TCM practitioners require the combination of clinical diagnosis and TCM syndrome differentiation. Moreover, most of the studies included in our analysis utilized enemas for treatment, which are administered in a significantly different manner compared to oral formulations, such as mesalazine. Therefore, it would have been difficult to perform allocation concealment and blinding. Second, there were fewer studies that reported loss to follow-up and subject withdrawals, and the possibility that a higher number of positive results was due to subjective factors of the patients and investigators could not be ruled out. Finally, all of the included studies had small sample sizes and did not provide details on sample size estimation, which decreased the statistical power of the analyses.

Based on the evidence of this meta-analysis, a network pharmacology approach was employed to illuminate the exact mechanism underlying the effects of Kushen on UC with DHAS with multiple components and targets. Based on the data retrieved from the SymMap and GeneCards database, *quercetin*, *luteolin*, *matrine*, *formononetin*, and *phaseolin* from Kushen were detected to play a key role in the treatment of DASH-UC with higher degrees. Nevertheless, targets of VEGFA, CASP3, IL6, EGFR, ESR1, MYC, CCND1, ERBB2, FOS, and MDM2 all played an important role in Kushen against UC with DHAS as the common genes with top 10° in the PPI networks. The above components and the cytokines of Kushen could perform critical roles in acute inflammation regulation/pro-inflammatory induction (Hong and Piao, 2018; Wu et al., 2018; Wang C. et al., 2019; Zhu et al., 2019). Therefore, the main bioactive components of Kushen may ameliorate UC with DHAS through internal intercellular mechanisms of pro-inflammatory cytokines, inflammatory pathways, and intestinal mucosa protection. By the Network Analyzer on C-T-P network, PI3K-Akt signaling was identified as the top core node of the pathways in Kushen against UC with DHAS with the greatest degrees. As an important signal transduction pathway, PI3K-Akt signaling is involved in the release of multiple pro-inflammatory cytokines, such as NF-κB, IL-6, and IL-17, which could control key cellular processes involved in the immune inflammatory response in UC once activated by Akt, indicating that Kushen may have an anti-inflammatory effect on UC with DHAS through PI3K-Akt signaling and its downstream pathways by acting on these target genes (Li et al., 2019).

To the best of our knowledge, this was the first attempt to track targets of UC with DHAS for further analysis of its interaction with herbal bioactive components. This analysis was based on a recently presented database, SymMap, which integrates TCM with modern medicine both at the molecular and phenotypic levels (Wang et al., 2019b; Wu et al., 2019). By integrating the meta-analysis and network pharmacology analysis, we successfully identified the efficacies of Kushen-based TCM formulations and the potential characteristic targets of Kushen against UC with DHAS.

However, besides quality of evidence, several other limitations are included in the present study. First, some possibilities may exist that the supplementary herbal medicines (prescribed for secondary syndromes treatment or toxicity reduction with small dosage) in the Kushen-based TCM formulations may exert positive effects. Second, pharmacology analysis was mainly employed to show a synergistic action of herbal chemical components acquired through existing databases rather than experimental verification, which needs further validation in future research.

## Conclusion

In conclusion, Kushen-based TCM formulations provide good efficacy and possess great potential in the treatment of UC. A major pathway (PI3K-Akt signaling pathway) of Kushen against DASH-UC was predicted from the results of network pharmacology analysis. This study presented an effective Chinese herbal medicine, Kushen, for UC with DHAS treatment, with a new prospect integrating meta-analysis and network pharmacology investigation for exploring the actual efficacies and mechanisms between diseases and TCM syndromes in more detail.

## Data Availability Statement

The original contributions presented in the study are included in the article/Supplementary Material, further inquiries can be directed to the corresponding author.

## Author Contributions

Conceptualization: ZT. Data analysis: MC and YD. Writing – original draft: MC. Writing – review and editing: MC and YD.

## Funding

This study was supported by the National Natural Science Foundation of China (No. 81673965). The funding agency was not involved in any aspect of the study design, data collection, data analysis, or manuscript writing.

## Conflict of Interest

The authors declare that the research was conducted in the absence of any commercial or financial relationships that could be construed as a potential conflict of interest.

## Abbreviations

DHAS, damp-heat accumulation syndrome; TCM, traditional Chinese medicine; UC, ulcerative colitis.

## References

[B1] Cao S.-Y. Ye S.-J. Wang W.-W. Wang B. Zhang T. Pu Y.-Q. (2019). Progress in active compounds effective on ulcerative colitis from Chinese medicines. Chin. J. Nat. Med. 17 (2), 81–102. 10.1016/s1875-5364(19)30012-3 30797423

[B2] Chen L. Shao J. Luo Y. Zhao L. Zhao K. Gao Y. (2020). An integrated metabolism *in vivo* analysis and network pharmacology in UC rats reveal anti-ulcerative colitis effects from Sophora flavescens EtOAc extract. J. Pharmaceut. Biomed. Anal. 186, 113306 10.1016/j.jpba.2020.113306 32371325

[B3] Chen X. He L. (2014). [Retention enema with TongGuan solution treat chronic ulcerative colitis randomized parallel group study]. J. Prac. Trad. Chinese Inter. Med. 28 (7), 36–38. 10.13729/j.issn.1671-7813.2014.07.16

[B4] Chen X. L. Wen Y. Wu Z. C. Zhang B. P. Hou Z. K. Xiao J. L. (2018). Development of a traditional Chinese medicine syndrome-specific scale for ulcerative colitis: the large intestine dampness-heat syndrome questionnaire. Evid. Based Complement. Alternat. Med. 2018, 4039019 10.1155/2018/4039019 30108653 PMC6077564

[B5] Chudnovskiy A. Mortha A. Kana V. Kennard A. Ramirez J. D. Rahman A. (2016). Host-Protozoan interactions protect from mucosal infections through activation of the inflammasome. Cell 167 (2), 444–456.e14. 10.1016/j.cell.2016.08.076 27716507 PMC5129837

[B6] Cumpston M. Li T. Page M. J. Chandler J. Welch V. A. Higgins J. P. (2019). Updated guidance for trusted systematic reviews: a new edition of the Cochrane Handbook for Systematic Reviews of Interventions. Cochrane Database Syst. Rev. 10, ED000142 10.1002/14651858.ED000142 31643080 PMC10284251

[B7] Ding Y. Chen M. Wang Q. Gao L. Feng Y. Wang S. (2020). Integrating pharmacology and microbial network analysis with experimental validation to reveal the mechanism of composite Sophora colon-soluble capsule against ulcerative colitis. Evid. Based. Complement. Alternat. Med. 2020, 9521073 10.1155/2020/9521073 32382313 PMC7189316

[B8] Eisenstein M. (2018). Ulcerative colitis: towards remission. Nature 563 (7730), S33 10.1038/d41586-018-07276-2 30405234

[B9] Fang R. Wu R. Zuo Q. Yin R. Zhang C. Wang C. (2018). Sophora flavescens containing-QYJD formula activates Nrf2 anti-oxidant response, blocks cellular transformation and protects against DSS-induced colitis in mouse model. Am. J. Chin. Med. [Epub ahead of print]. 10.1142/S0192415X18500829 PMC8111688 30284461

[B10] Gan D. Xu X. Chen D. Feng P. Xu Z. (2019). Network pharmacology-based pharmacological mechanism of the Chinese medicine rhizoma drynariae against osteoporosis. Med. Sci. Monit. 25, 5700–5716. 10.12659/MSM.915170 31368456 PMC6688518

[B11] Gong Y. Zha Q. Li L. Liu Y. Yang B. Liu L. (2012). Efficacy and safety of Fufangkushen colon-coated capsule in the treatment of ulcerative colitis compared with mesalazine: a double-blinded and randomized study. J. Ethnopharmacol. 141 (2), 592–598. 10.1016/j.jep.2011.08.057 21911045

[B12] Guo W. Huang J. Wang N. Tan H. Y. Cheung F. Chen F. (2019). Integrating network pharmacology and pharmacological evaluation for deciphering the action mechanism of herbal formula zuojin pill in suppressing hepatocellular carcinoma. Front. Pharmacol. 10, 1185 10.3389/fphar.2019.01185 31649545 PMC6795061

[B13] He H. Shen H. Zhen K. Gu P. Zhu L. Liu Y. (2012). [Observation of the curative effect of qingchang huashi recipe for treating active ulcerative colitis of inner-accumulation of damp-heat syndrome]. Zhongguo Zhong Xi Yi Jie He Za Zhi 32 (12), 1598–1601. 23469594

[B14] Holleran G. Scaldaferri F. Gasbarrini A. Curro D. (2020). Herbal medicinal products for inflammatory bowel disease: a focus on those assessed in double-blind randomised controlled trials. Phytother Res. 34 (1), 77–93. 10.1002/ptr.6517 31701598

[B15] Hong Z. Piao M. (2018). Effect of quercetin monoglycosides on oxidative stress and gut microbiota diversity in mice with dextran sodium sulphate-induced colitis. BioMed Res. Int. 2018, 8343052 10.1155/2018/8343052 30539022 PMC6260418

[B16] Langhorst J. Wulfert H. Lauche R. Klose P. Cramer H. Dobos G. J. (2015). Systematic review of complementary and alternative medicine treatments in inflammatory bowel diseases. J. Crohns Colitis 9 (1), 86–106. 10.1093/ecco-jcc/jju007 25518050

[B17] Levy M. Thaiss C. A. Zeevi D. Dohnalova L. Zilberman-Schapira G. Mahdi J. A. (2015). Microbiota-modulated metabolites shape the intestinal microenvironment by regulating NLRP6 inflammasome signaling. Cell 163 (6), 1428–1443. 10.1016/j.cell.2015.10.048 26638072 PMC5665753

[B18] Li N. Sun W. Zhou X. Gong H. Chen Y. Chen D. (2019). Dihydroartemisinin protects against dextran sulfate sodium-induced colitis in mice through inhibiting the PI3K/AKT and NF-kappaB signaling pathways. BioMed Res. Int. 2019, 1415809 10.1155/2019/1415809 31781591 PMC6875009

[B19] Lin Y. H. Luck H. Khan S. Schneeberger P. H. H. Tsai S. Clemente-Casares X. (2019). Aryl hydrocarbon receptor agonist indigo protects against obesity-related insulin resistance through modulation of intestinal and metabolic tissue immunity. Int. J. Obes. (Lond) 43 (12), 2407–2421. 10.1038/s41366-019-0340-1 30944419 PMC6892742

[B20] Lu Y. H. Cong L. L. (2012). [Study on the Chinese medical syndrome distribution of ulcerative colitis]. Zhongguo Zhong Xi Yi Jie He Za Zhi 32(4), 450–454. 22803420

[B21] Magro F. Gionchetti P. Eliakim R. Ardizzone S. Armuzzi A. Barreiro-de Acosta M. (2017). Third European evidence-based consensus on diagnosis and management of ulcerative colitis. Part 1: definitions, diagnosis, extra-intestinal manifestations, pregnancy, cancer surveillance, surgery, and ileo-anal pouch disorders. J. Crohns Colitis 11 (6), 649–670. 10.1093/ecco-jcc/jjx008 28158501

[B22] Martin A. Ochagavia M. E. Rabasa L. C. Miranda J. Fernandez-de-Cossio J. Bringas R. (2010). BisoGenet: a new tool for gene network building, visualization and analysis. BMC Bioinf. 11, 91 10.1186/1471-2105-11-91 PMC3098113 20163717

[B23] Naganuma M. Mizuno S. Nanki K. Sugimoto S. Kanai T. (2016). Recent trends and future directions for the medical treatment of ulcerative colitis. Clin. J. Gastroenterol 9 (6), 329–336. 10.1007/s12328-016-0686-z 27699641

[B24] Naganuma M. Sugimoto S. Mitsuyama K. Kobayashi T. Yoshimura N. Ohi H. (2018). Efficacy of indigo naturalis in a multicenter randomized controlled trial of patients with ulcerative colitis. Gastroenterology 154 (4), 935–947. 10.1053/j.gastro.2017.11.024 29174928

[B25] Naganuma M. (2019). Treatment with indigo naturalis for inflammatory bowel disease and other immune diseases. Immunol. Med. 42 (1), 16–21. 10.1080/25785826.2019.1599158 31034341

[B26] Ng S. C. Bernstein C. N. Vatn M. H. Lakatos P. L. Loftus E. V. Jr. Tysk C. (2013). Geographical variability and environmental risk factors in inflammatory bowel disease. Gut 62 (4), 630–649. 10.1136/gutjnl-2012-303661 23335431

[B27] Ng S. C. Leung W. K. Shi H. Y. Li M. K. Leung C. M. Ng C. K. (2016). Epidemiology of inflammatory bowel disease from 1981 to 2014: results from a territory-wide population-based registry in Hong Kong. Inflamm. Bowel Dis. 22 (8), 1954–1960. 10.1097/MIB.0000000000000846 27416041

[B28] Ordas I. Eckmann L. Talamini M. Baumgart D. C. Sandborn W. J. (2012). Ulcerative colitis. Lancet 380 (9853), 1606–1619. 10.1016/S0140-6736(12)60150-0 22914296

[B29] Qu H. (2011). [Clinical observation of combined sulfasalazine with retention enema of “Baiji Kusen Decoction’’ in treating ulcerative colitis]. Shanghai J. TCM 45 (12), 56–57. 10.16305/j.1007-1334.2011.12.038

[B30] Reimand J. Isserlin R. Voisin V. Kucera M. Tannus-Lopes C. Rostamianfar A. (2019). Pathway enrichment analysis and visualization of omics data using g:Profiler, GSEA, Cytoscape and EnrichmentMap. Nat. Protoc. 14 (2), 482–517. 10.1038/s41596-018-0103-9 30664679 PMC6607905

[B31] Ru J. Li P. Wang J. Zhou W. Li B. Huang C. (2014). TCMSP: a database of systems pharmacology for drug discovery from herbal medicines. J. Cheminf. 6, 13 10.1186/1758-2946-6-13 PMC4001360 24735618

[B32] Sandborn W. J. Su C. Sands B. E. D'Haens G. R. Vermeire S. Schreiber S. (2017). Tofacitinib as induction and maintenance therapy for ulcerative colitis. N. Engl. J. Med. 376 (18), 1723–1736. 10.1056/NEJMoa1606910 28467869

[B33] Sharma D. Malik A. Guy C. S. Karki R. Vogel P. Kanneganti T. D. (2018). Pyrin inflammasome regulates tight junction integrity to restrict colitis and tumorigenesis. Gastroenterology 154 (4), 948–964.e8. 10.1053/j.gastro.2017.11.276 29203393 PMC5847456

[B34] Tong Z. Q. Yang B. Chen B. Y. Zhao M. L. (2010). A multi-center, randomized, single-blind, controlled clinical study on the efficacy of composite sophora colon-soluble capsules in treating ulcerative colitis. Chin. J. Integr. Med. 16 (6), 486–492. 10.1007/s11655-010-0562-5 21110172

[B35] Tong Z. Q. Yang B. Tong X. Y. (2011). [A multi-center randomized double-blinded, placebo-controlled clinical study on efficacy of composite sophora colon-soluble capsules in treating ulcerative colitis of internal dampness-heat accumulation syndrome type]. Zhongguo Zhong Xi Yi Jie He Za Zhi 31 (2), 172–176. 21425568

[B36] Wang C. Li W. Wang H. Ma Y. Zhao X. Zhang X. (2019). Saccharomyces boulardii alleviates ulcerative colitis carcinogenesis in mice by reducing TNF-alpha and IL-6 levels and functions and by rebalancing intestinal microbiota. BMC Microbiol. 19 (1), 246 10.1186/s12866-019-1610-8 31694526 PMC6836350

[B37] Wang N. Yang B. Zhang J. Zheng Y. Wang S. Zhang X. (2020). Metabolite profiling of traditional Chinese medicine XIAOPI formula: an integrated strategy based on UPLC-Q-Orbitrap MS combined with network pharmacology analysis. Biomed. Pharmacother. 121, 109569 10.1016/j.biopha.2019.109569 31739163

[B38] Wang P. Dai L. Zhou W. Meng J. Zhang M. Wu Y. (2019). Intermodule coupling analysis of huang-lian-jie-du decoction on stroke. Front. Pharmacol. 10, 1288 10.3389/fphar.2019.01288 31772561 PMC6848980

[B39] Wang T. Streeter H. Wang X. Purnama U. Lyu M. Carr C. (2019). A network pharmacology study of the multi-targeting profile of an antiarrhythmic Chinese medicine xin su ning. Front. Pharmacol. 10, 1138 10.3389/fphar.2019.01138 31607935 PMC6774044

[B40] Wang X. Saud S. M. Zhang X. Li W. Hua B. (2019a). Protective effect of Shaoyao Decoction against colorectal cancer via the Keap1-Nrf2-ARE signaling pathway. J. Ethnopharmacol. 241, 111981 10.1016/j.jep.2019.111981 31146002

[B41] Wang X. Yu H. Bai C. Xu J. N. Zhen J. H. Gu X. H. (2019b). [Pharmacological mechanism of Shenghua Decoction in treatment of abdominal pain based on network pharmacology]. Zhongguo Zhong Yao Za Zhi 44 (10), 2124–2130. 10.19540/j.cnki.cjcmm.20190218.003 31355571

[B42] Wu D. Wu K. Zhu Q. Xiao W. Shan Q. Yan Z. (2018). Formononetin administration ameliorates dextran sulfate sodium-induced acute colitis by inhibiting NLRP3 inflammasome signaling pathway. Mediat. Inflamm. 2018, 3048532 10.1155/2018/3048532 PMC5817291 29507526

[B43] Wu Y. Zhang F. Yang K. Fang S. Bu D. Li H. (2019). SymMap: an integrative database of traditional Chinese medicine enhanced by symptom mapping. Nucleic Acids Res. 47 (D1), D1110–D1117. 10.1093/nar/gky1021 30380087 PMC6323958

[B44] Xu M. Duan X. Y. Chen Q. Y. Fan H. Hong Z. C. Deng S. J. (2019). Effect of compound sophorae decoction on dextran sodium sulfate (DSS)-induced colitis in mice by regulating Th17/Treg cell balance. Biomed. Pharmacother. 109, 2396–2408. 10.1016/j.biopha.2018.11.087 30551499

[B45] Ye B. Shen H. Lu Y. Wang Y. Q. (2010). Clinical observations on 100 cases of ulcerative colitis treated with the method of clearing away heat, expelling dampness, promoting blood circulation and healing ulcer. J. Tradit. Chin. Med. 30 (2), 98–102. 10.1016/s0254-6272(10)60022-2 20653164

[B46] Zhang J. Wang X. Chen H. (2015). [Integrated traditional Chinese medicine-W est medicine for the therapy of chronic ulcerative colitis report]. Med. Recapitulate 21 (14), 2683–2684+2689. 10.3969/j.issn.1006-2084.2015.14.074

[B47] Zhang S. Shen H. Zhen K. Ye B. (2017). Expert consensus on TCM diagnosis and treatment of ulcerative colitis (2017). China J, TCM Phar. 32 (8), 3585–3589.

[B48] Zhao J. (2017). [ClinicaI research on compound kushen enteric capsules in the treatment of UIcerative colitis with dampness-heat accumulated in interior syndrome]. Chinese M Dis. Edu. 15 (13), 48–49. 10.3969/j.issn.1672-2779.2017.13.022

[B49] Zhu L. Gu P. Shen H. (2019). Gallic acid improved inflammation via NF-kappaB pathway in TNBS-induced ulcerative colitis. Int. Immunopharm. 67, 129–137. 10.1016/j.intimp.2018.11.049 30544066

